# Tobacco regulatory compliance with STAKE Act age-of-sale signage among licensed tobacco retailers across diverse neighborhoods in Southern California

**DOI:** 10.18332/tid/91846

**Published:** 2018-06-14

**Authors:** Steve Sussman, Tess Boley Cruz, Sabrina L. Smiley, Chih-Ping Chou, Jennifer B. Unger, Natalie Kintz, Yaneth L. Rodriguez, Rosa Barahona, Brianna A. Lienemann, Mary Ann Pentz, Jonathan Samet, Lourdes Baezconde-Garbanati

**Affiliations:** 1 USC Tobacco Center for Regulatory Sciences in Vulnerable Populations, Keck School of Medicine, University of Southern California, United States; 2 Colorado School of Public Health, University of Colorado, Aurora, United States

**Keywords:** tobacco, age-of-sale signs, compliance, licensed tobacco retailers, race/ethnicity

## Abstract

**INTRODUCTION:**

The California Stop Tobacco Access to Kids Enforcement (STAKE) Act requires licensed tobacco retailers to post minimum age-of-sale signage at the point of sale. This study investigated STAKE Act compliance in licensed tobacco retailers across four racial/ethnic communities in Southern California.

**METHODS:**

The sample consisted of 675 licensed tobacco retailers (excluding chain store supermarkets and pharmacies) randomly selected based on zip codes from predominantly non-Hispanic White (n=196), African American (n=193), Hispanic/Latino (n=186), and Korean American (n=100) communities. A protocol for assessing signage was completed at each store by community health workers (*promotoras de salud*). The law changed from a minimum age of 18 to 21 years (Tobacco 21) during data collection, as of 9 June 2016. Differences in signage compliance were evaluated before and after changes in the State law.

**RESULTS:**

Overall, 45% of the stores were compliant with posting the required age-of-sale signage (which varied in minimum age by date of collection); 14% of stores did not have any store interior age-of-sale signs, and 41% of stores had some type of age-of-sale sign but were not compliant with the STAKE Act (e.g. 29.5% of the stores had non-compliant tobacco industry We Card signs but not STAKE Act signs). Stores observed after the 2016 implementation of Tobacco 21 had significantly lower STAKE Act signage compliance rates (38.6%) compared to stores observed before the change in the State law (70.9%) (z=6.8623, p<0.001). The difference in STAKE Act sign compliance between stores located in AA communities (16.9%) and stores located in NHW communities (41.5%) observed within the first three months after the change in law was statistically significant (χ^2^(1)=20.098, p<0.001).

**CONCLUSIONS:**

Findings suggest the need for prompt, educational outreach to licensed tobacco retailers on age-of-sale signage changes, multiple compliance checks, and enforcement.

## INTRODUCTION

Use of age-of-sale signs in licensed tobacco retailers in the United States is a method for limiting youth access to tobacco products^1^. While a few studies^2,3^ have found no difference in tobacco sales to minors in stores, with vs without age-of-sale signs, a recent review indicates that, in general, posting age-of-sale signage increases the likelihood that licensed tobacco retailers will check the identification of potentially underage consumers4, resulting in a 2% reduction in tobacco use prevalence among youth^5^. Because age-of-sale signs in licensed tobacco retailers can prevent tobacco sales to minors and discourage minors from attempting to purchase tobacco, it is important to assess compliance with laws that mandate such signage. Indeed, a few recent studies^4,6,7^ have found high compliance, ranging from 81.8 to 87.1%. However, there is a paucity of such studies, particularly in socioeconomically disadvantaged and racial/ethnic diverse communities.

The initial 1994 California Stop Tobacco Access to Kids Enforcement (STAKE) Act required retailers to post at each point of purchase, an age-of-sale sign that includes a telephone number to report failures to check identification for tobacco purchases^8^. With the amendment of California Senate Bill (SB x2–7) the Tobacco Products: Minimum Legal Age Bill (Tobacco 21) in May 2016, the minimum tobacco purchase age in California became 21 years; although it was 18 years when that law was initially enacted. California’s required signage under the STAKE Act is a ‘1–800–5 ASK–4–ID’ warning sign that indicates that sale of tobacco products to persons under a fixed age is illegal (except for military personnel). The minimum age law changed during data collection in the present study, which spanned January 2016 to April 2017. STAKE Act signs are distributed by the State of California to California retailers when they apply for or renew their tobacco retail licenses, and they are the official signage the State makes available to all California licensed tobacco retailers through the Tobacco Education Clearinghouse of California^9^.

The tobacco industry also began to provide minimum age signage for retailers in 1995 with its ‘We Card’ campaign, begun by the National Coalition for Responsible Tobacco Retailing and Philip Morris^10^. This signage is not endorsed by the State of California, but is endorsed by multiple tobacco companies and other organizations^11^ (http://www.wecard.org/supporting-members; accessed 11–5–2017). It has been asserted, based on an analysis of tobacco industry documents, that this program was established to improve the industry’s image and reduce public regulation and enforcement^8^. This signage by the tobacco industry may have confused some licensed tobacco retailers. It became necessary in 2002, for the California Attorney General’s Office to have the Coalition for Responsible Tobacco Retailing and Philip Morris revise the information and materials they distributed to retailers to communicate that the We Card sign did not comply with State law^12^. Tobacco companies have also distributed other types of signage not endorsed by the State of California, including ‘Buying tobacco for minors could cost you. It’s not just wrong. It’s illegal’ (Philip Morris, USA); and a calendar sign stating ‘You must have been born on or before today’s date [1995]’ (Reynolds American, branded with a Natural American Spirit logo). However, there is little evidence of effectiveness of tobacco industry signs in reducing sales to minors^8,10^.

Despite California’s strong control laws on tobacco, there are few empirical studies about age-of-sale sign compliance among licensed tobacco retailers^12^, particularly pertaining to stores in various racial/ethnic communities^13-15^. This study investigated the presence of interior display of age-of-sale signs in licensed tobacco retailers located in low-income, predominantly African American (AA), Korean American (KA), Hispanic/Latino (H/L) and Non-Hispanic White (NHW) communities in Southern California communities. These communities were selected because of historical patterns of targeting by tobacco industry promotions at the point-of-sale and relatively high combustible tobacco product use prevalence^13-16^. For these reasons, age-of-sale sign compliance would be important to maintain.

## METHODS

### Licensed tobacco retailer sample

The target sample was 700 stores. The obtained sample consisted of 675 Los Angeles County stores that were licensed to sell tobacco; stores were smaller independent stores that included markets, convenience stores with or without a gas station, liquor stores and tobacco stores located in communities that were predominantly AA (n=193 stores), H/L (n=186 stores), NHW (n=196 stores) or KA (n=100 stores). Independent and small licensed tobacco retailers were included while pharmacies, big chain markets/supermarkets, as well as emerging vape shops were excluded from this study. Prior research has shown that independent and smaller licensed tobacco retailers are less likely to ask for identification compared with chain stores^17^ and have more tobacco advertising^18^. The selection of the neighborhoods and licensed tobacco retailers in this study is explained in detail by Baezconde-Garbanati et. al.^19^ and summarized below.

First, zip codes in Los Angeles County with a median or below median household income were ranked by percentage of race/ethnicity for each specific community. The number of available zip codes that met the criteria for each community differed slightly, with seven zip codes for KA, 14 for AA, 14 for HL, and 32 for NHW. To be consistent across all communities of focus, it was decided to select up to 15 zip codes available from each community identified. This criterion most affected the NHW community sample, as there were 32 eligible zip codes available. All other communities had less than 15 zip codes that met the criteria. Therefore, all the zip codes were kept in those communities. We exhausted all possible stores in the top 15 zip codes for the NHW community before reaching our intended sample size. The strategy was to select the next eligible zip codes, until we reached our sample size. We collected data from 21 zip codes out of the possible 32 zip codes in the NHW community. From the 296 zip codes in Los Angeles County that have licensed tobacco retailers, data were collected for 56 zip codes for this study (19%).

Next, licensed tobacco retailers were randomly selected from ranked zip codes using the California Department of Tax and Fee Administration (formerly called the Board of Equalization) list of stores with tobacco retailer licenses^20^. The number of stores selected in each zip code was based in proportion to the race/ethnicity ranking of each community. There were approximately 11600 stores licensed to sell tobacco in Los Angeles County. Approximately 10200 of these stores were eligible under our criteria, and 2556 of the eligible stores were in the selected zip codes for this study (22% of all licensed stores in Los Angeles County). Additional stores were deemed ineligible if, after we visited them, a store no longer sold tobacco, no longer existed, or was a pharmacy or big chain supermarket. A total of 1480 licensed tobacco retailers were randomly selected and visited; 310 were deemed ineligible based on the above criteria. Thus, we visited 1170 eligible stores, to attempt to interview persons at 700 stores. Only 29% declined to participate; we did not enroll 130 licensed tobacco retailers because we met our sample size target of 700. In total 700 retailers were interviewed, of which 679 were licensed tobacco retailers that allowed an observation to be conducted but because of missing data only 675 of these were included in this STAKE Act signage study. We estimate that our sample represents 21% of the licensed tobacco retailers that sell tobacco within the zip codes selected for all communities, and 6% of all licensed tobacco retailers in Los Angeles County^20^.

### Data collection

Store owners, managers or clerks/cashiers consented to complete a 30-minute interview survey and to allow store naturalistic observation to be conducted by two separate community health workers (CHWs) during the same store visit (one CHW conducted the interview survey and the second CHW conducted the observation). We ensured that at least one of the two CHWs, at each store visit, represented the predominant race/ethnicity of the community and spoke the language of the retailers. Stores that agreed to be part of the study were compensated with $125 in gift cards and received a leave-behind packet containing tobacco fact sheets about the Food and Drug Administration (FDA) tobacco regulations and sales to minors (available in English, Spanish and Korean).

The store observation instrument is an adapted version of the Standardized Tobacco Assessment for Retail Settings (STARS) observation tool^21^. This tool reliably documents the presence of selected tobacco products and store characteristics, and was adapted by our study team to record indoor and outdoor signage on minimum age limits for tobacco sales. Indoor (legal) signage was the focus of this study. The human subjects protocol for this research was approved by the University of Southern California Institutional Review Board.

Data collection occurred from January 2016 to April 2017. Data collectors observed the presence of three types of age-of-sale signs inside the store: 1) the mandated age-of-sale sign by the STAKE Act at the point of purchase of tobacco products, 2) the tobacco industry We Card sign, and 3) other (with an open text field to describe what ‘other’ types of age-of-sales signs were present in the store). The ‘other’ text field responses were then coded either as other Philip Morris (‘Buying tobacco for minors could cost you. It’s not just wrong. It’s illegal’), Natural American Spirit (‘You must have been born on or before today’s date [1995]’), homemade signage, other California sign (e.g. California ID verification tip sheet or California We Check ID Window Cling), or other (anything else that did not fit into any of these categories).

In addition to the presence and location of age-of-sale signage, the minimum age on the sign was documented (whether the sign listed an 18+ or 21+ minimum age). This allowed us to determine if a store had the appropriate STAKE Act signage or inappropriate signage based on the date of observation. Inappropriate STAKE Act signs are those that listed the minimum age as 18 years instead of 21 years for stores that were observed after the Tobacco 21 law took effect in California.

Twenty-five per cent of all the stores in the sample were observed by one additional CHW to ensure inter-rater reliability (in these cases, two CHWs observed the store at the same time). A Kappa statistic was used to test the inter-rater reliability for the STAKE Act and We Card signage. The Kappa statistic for the STAKE Act signage was 0.73 (moderate agreement) and for the We Card was 0.84 (strong agreement)^22^. We had taken photos at the point of sale for 82% of the stores. We examined the photos to help reconcile disagreements.

### Data analysis

Frequency distributions were used to evaluate the number of stores that were compliant, and what other types of age-of-sale signs were displayed in stores that were not compliant with the STAKE Act. A store was considered compliant with STAKE Act signage if it had an appropriate STAKE Act sign posted inside the store at the point-of-sale, depending on the date of observation (an 18+ STAKE Act sign before, and a 21+ STAKE Act sign after, 9 June 2016). Tobacco 21 was implemented in the middle of the study, which was not originally designed to examine differences across all four communities before and after the change in law. Due to the survey schedule, most of the H/L location retailers were observed before the change in law, while all the KA location retailers were observed more than 3 months after the law change. Since the communities were not observed in a balanced timeframe before and after the change in law, it was not possible to examine changes in compliance rates with the Stake Act across all communities before and after the change in law, or over time after the change in law. However, a Z-test was conducted to test the proportions of compliance between stores observed before and after the change in law to evaluate whether fewer stores were compliant after the change in law. Furthermore, frequency distributions and cross-tabulations were used for basic statistical descriptions of community compliance rates for three time periods: 1) before (6/9/16 to 9/8/16) the Stake Act; 2) the first three months (9/9/16 to 12/8/16) following the Stake Act; and 3) more than 3 months following the Stake Act (12/9/16 to 4/6/17). In addition, since the majority of stores from the AA and NHW communities were observed within the first three months after the law change (6/9/16 to 9/8/16), there was a sufficient number of licensed tobacco retailers to compare differences between these two communities during that period. Pearson’s chi-squared statistic was used to assess compliance between AA and NHW communities during the first three months following the Stake Act. An α<0.05 was used for significance in all statistical analyses and the data were analyzed^23^ using SAS v 9.4.

## RESULTS

Overall, 45% of the stores were compliant with the required STAKE Act age-of-sale sign posted (Table 1), 14% of the stores did not have any store interior age-of-sale signs, and 41% had some type of age-of-sale sign but were not compliant with the STAKE Act. Table 2 provides the frequencies of non-STAKE Act age-of-sale signs that were displayed in the non-compliant stores. For example, 29.5% of retailers that did not have the appropriate STAKE Act sign had the tobacco industry We Card sign, and 10.6% had homemade signs posted inside the store (Table 2).

**Table 1:** STAKE Act signage compliance and other signage by community: Los Angeles County, California ( 01/27/16–04/6/17 )

*Community*	*Compliant with STAKE Act Signage N (%)*	*Other Signs Not Compliant N (%)*	*No Age-of-Sale Signs at all Not Compliant N (%)*
AA (n=193)	55 (28.5)	110 (57.0)	28 (14.5)
KA (n=100)	60 (60.0)	26 (26.0)	14 (14.0)
H/L (n=186)	108 (58.1)	50 (26.9)	28 (15.1)
NHW (n=196)	83 (42.3)	91 (46.4)	22 (11.2)
Total (n=675)	306 (45.3)	277 (41.0)	92 (13.6)

**Table 2:** Frequency of Other Age-of-Sale Signs displayed in the interior of stores that were not compliant with the STAKE Act Signage ( 18+ sign before 9 June 2016; 21+ sign on or after 9 June 2016 )

*Community*	*We Card N (%)*	*Other Philip Morris N (%)*	*Natural America Spirit N (%)*	*Handmade sign N (%)*	*Other CA sign N (%)*	*Other N (%)*	*No sign N (%)*
AA (N=138)	34 (24.6)	10 (7.3)	6 (4.4)	15 (10.9)	27 (19.6)	20 (14.5)	28 (20.3)
KA (N=40)	11 (27.5)	8 (20.0)	2 (5.0)	4 (10.0)	7 (17.5)	4 (10.0)	14 (35.0)
HL (N=78)	20 (25.6)	12 (15.4)	1 (1.3)	4 (5.1)	12 (15.4)	7 (9.0)	28 (35.9)
NHW (N=113)	44 (38.9)	22 (19.5)	10 (8.9)	16 (14.2)	12 (10.6)	12 (10.6)	22 (19.5)
Total (N=369)	109 (29.5)	52 (14.1)	19 (5.2)	39 (10.6)	58 (15.7)	43 (11.7)	92 (24.9)

We Card: sign developed by the National Coalition for Responsible Tobacco Retailing and Philip Morris. Other Philip Morris: ‘Buying tobacco for minors could cost you. It’s not just wrong. It’s illegal’. Natural American Spirit: ‘You must have been born on or before today’s date [1995]’. Homemade sign: Any sign that the retailers created on their own. Other CA sign: California ID verification tip sheet or California We Check ID Window Cling. Other: Anything else that did not fit into any of the above categories (e.g. ‘The sale of tobacco products is prohibited to persons under 21’, ‘We-Check ID Under 30’).

Twenty-five per cent of the licensed tobacco retailers were observed before California implemented Tobacco 21 (n=141); 75% were observed after the minimum age law was changed (n=534). Licensed tobacco retailers observed after the change in the STAKE Act law exhibited a significantly lower compliance rate (38.6%) compared to stores observed before the change in the STAKE Act law (70.9%) (z=6.8623, p<0.001). Table 3 shows that compliance rates for stores observed within the first three months after the change in law were lower (26.7%) than other time periods. Considered over all time points after the law changed, the licensed tobacco retailer compliance rate was 42.3% in the NHW and 24.6% in the AA communities. Observed within the first three months after the change in law, STAKE Act sign compliance was much lower at licensed tobacco retailers in AA communities (16.9% compliance) than in NHW communities (41.5%; χ^2^(1)=20.098, p<0.001). However, the compliance rate was much higher in those AA community licensed tobacco retailers that were observed after the first three months (66.7%). Compliance rates for stores in KA communities that were observed after the change in law were higher overall (60.0%) compared to those of AA (24.6%) and NHW (42.3%) communities; no KA stores were observed within the first three months following the change in law. While there were too few post-law retailers to engage in a formal analysis, retailers in H/L and AA communities showed a similar pattern: retailers observed immediately after the change in the law had lower compliance rates than the retailers observed after the first 3 months.

**Table 3:** STAKE Act compliance rates observed from stores across different communities before and after the change in law

	*Overall Compliance*		*STAKE Act 18+ Pre-Tobacco 21*				*STAKE Act 21+ Post-Tobacco 21*			
	*Entire Study 01/27/16–04/06/17*		*01/27/16–06/08/17*		*06/09/16–04/06/17*		*≤ 3 months 06/09/16–09/08/16*		*>3 months 09/09/16–04/06/17*	
	*Compliant*	*Total*	*Compliant*	*Total*	*Compliant*	*Total*	*Compliant*	*Total*	*Compliant*	*Total*

*Community*	*N (%)*	*N*	*N (%)*	*N*	*N (%)*	*N*	*N (%)*	*N*	*N (%)*	*N*
AA	55 (28.5)	193	12 (66.7)	18	43 (24.6)	175	25 (16.9)*	148	18 (66.7)	27
KA	60 (60.0)	100	-	0^+^	60 (60.0)	100	-	0^+^	60 (60.0)	100
H/L	108 (58.1)	186	88 (71.5)	123	20 (31.8)	63	8 (18.2)	44	12 (63.6)	19
NHW	83 (42.3)	196	-	0^+^	83 (42.3)	196	51 (41.5)*	123	32 (43.8)	73
Total	306 (45.3)	675	100 (70.9)	141	206 (38.6)	534	84 (26.7)	315	122 (55.7)	219

* Association between AA and NHW stores observed within the first three months after Tobacco 21 was statistically significant (χ^2^(1)=20.098, p<0.001), where AA stores were less likely to be compliant than NHW stores.

+ No stores were observed in this interval.

## DISCUSSION

Considered across all time points, slightly over half of licensed tobacco retailers in four ethnic neighborhoods in Southern California were not compliant with STAKE Act regulations on signage required by law. STAKE Act compliance was higher (70.9%) among the retailers observed prior to Tobacco 21 implementation, which is consistent with previous report findings^24^ for the years 2009 to 2016. The compliance rates for stores observed after the change in law (38.6%) were comparable to STAKE Act compliance rates^24^ in the period 1998–2001. Most stores attempt to comply with the spirit of the STAKE Act regulations by placing the correct age-of-sale signs in their stores. However, 41% of stores posted other signs that do not meet the STAKE Act signage requirements. Licensed tobacco retailers might be unaware that homemade signs or signs distributed by the tobacco industry do not meet the STAKE Act requirements. Overall, 13.6% of retailers had no age-of-sale signs.

The impact of Tobacco 21 laws appears to generalize equally across different race/ethnicity communities in some recent work^25^. In our study, initial non-compliance decreased rapidly in stores located in AA communities relative to stores in NHW communities upon implementation of the new minimum age policy. Our findings, however, also do suggest the need for better education of retailers on age-of-sale sign compliance with attention to the timing of that education with regular follow-ups for several months after there has been a change in the law.

The strength of this study is that we examine a large sample of licensed tobacco retailers in diverse communities in Los Angeles County, which provides a reasonable gauge of age-of-sale sign policy compliance. However, a limitation of the study is that it was cross-sectional (i.e. different stores were observed at different time points within the same ethnic locations) and different ethnic locations were not observed systematically prior to and after implementation of the new Tobacco 21 policy. The study was not designed to look at differences across all four communities before and after the change in law, since communities were observed at different time points, but there were some interesting results that warrant further investigation. Another limitation of this study is that the non-cooperation rate was 29%. One may speculate that retailers who refused participation may be less likely to comply with signage and other requirements. Therefore, the study could underestimate non-compliance.

Prior to Tobacco 21, and after a 22-year period (1994–2016), approximately 30% of stores did not comply with the 18-year old minimum age STAKE Act signage law. Compliance with the previous law was similar across ethnic neighborhoods (specifically stores in AA and HL communities). However, one may speculate based on the results of the current study that the time required to come into compliance with a new law might vary across racial/ethnic neighborhoods. Future studies could better evaluate what accounts for differences in compliance in the various race/ethnic communities (e.g. by utilizing a repeated measures design).

Tobacco control agencies could provide educational outreach and spot-checks of individual retailers to reduce non-compliance in the time period immediately before and after the law changes, with periodic checks thereafter. Sending retailers new STAKE Act signs each year, and possibly color coding the year of the sign (like car registration tags for license plates), might also help increase compliance. Future studies are needed to explore barriers to compliance. These issues need to be explored as well in other regions of California and in other States.

## CONCLUSIONS

It is important to note that 70% of stores complied with STAKE Act signage requirements about tobacco sales to minors before the age-of-sale changed from 18 to 21 years of age. However, while only suggestive, this level of compliance appeared to have dropped significantly in the time shortly after changes in the age law, with a greater drop in AA communities compared to NHW communities. Findings suggest the need for prompt and better education of retailers to improve age-of-sale sign compliance at times of change in the law, and multiple compliance checks for new signage afterward.
